# The Paget Trial: A Multicenter, Observational Cohort Intervention Study for the Clinical Efficacy, Safety, and Immunological Response of Topical 5% Imiquimod Cream for Vulvar Paget Disease

**DOI:** 10.2196/resprot.7503

**Published:** 2017-09-06

**Authors:** Michelle van der Linden, Kim Meeuwis, Colette van Hees, Eleonora van Dorst, Johan Bulten, Tjalling Bosse, Joanna IntHout, Dorry Boll, Brigitte Slangen, Manon van Seters, Marc van Beurden, Mariëtte van Poelgeest, Joanne de Hullu

**Affiliations:** ^1^ Department of Obstetrics and Gynaecology Radboud University Medical Center Nijmegen Netherlands; ^2^ Department of Dermatology Radboud University Medical Center Nijmegen Netherlands; ^3^ Department of Dermatology Erasmus Medical Center Rotterdam Netherlands; ^4^ Department of Gynaecology University Medical Center Utrecht Utrecht Netherlands; ^5^ Department of Pathology Radboud University Medical Center Nijmegen Netherlands; ^6^ Department of Pathology Leiden University Medical Center Leiden Netherlands; ^7^ Department for Health Evidence Radboud Institute for Health Sciences Nijmegen Netherlands; ^8^ Department of Gynaecology Catharina Hospital Eindhoven Netherlands; ^9^ Department of Gynaecology Maastricht University Medical Center Maastricht Netherlands; ^10^ Department of Obstetrics & Gynaecology Worchester Royal Hospital Worchester United Kingdom; ^11^ Department of Gynaecology Netherlands Cancer Institute Antoni van Leeuwenhoek Hospital Amsterdam Netherlands; ^12^ Department of Gynaecology Leiden University Medical Center Leiden Netherlands

**Keywords:** Paget disease, extramammary Paget disease, vulvar Paget disease, imiquimod

## Abstract

**Background:**

Vulvar Paget disease is a rare skin disorder, which is most common in postmenopausal Caucasian women. They usually present with an erythematous plaque that may show fine or typical “cake icing” scaling or ulceration that may cause itching, pain, irritation, or a burning sensation. Although most cases are noninvasive, vulvar Paget disease may be invasive or associated with an underlying vulvar or distant adenocarcinoma. The histological evidence of so-called “Paget cells” with abundant pale cytoplasm in the epithelium confirms the diagnosis. The origin of these Paget cells is still unclear. Treatment of choice is wide local excision with negative margins. Obtaining clear surgical margins is challenging and may lead to extensive and mutilating surgery. Even then, recurrence rates are high, ranging from 15% to 70%, which emphasizes the need for new treatment options. A number of case reports, retrospective case series, and one observational study have shown promising results using the topical immune response modifier imiquimod.

**Objective:**

This study aims to investigate the efficacy, safety, and immunological response in patients with noninvasive vulvar Paget disease using a standardized treatment schedule with 5% imiquimod cream.

**Methods:**

Topical 5% imiquimod cream might be an effective and safe treatment alternative for vulvar Paget disease. The Paget Trial is a multicenter observational cohort study including eight tertiary referral hospitals in the Netherlands. It is ethically approved by the Medical-Ethical Committee of Arnhem-Nijmegen and registered in the Central Committee on Research Involving Human Subjects (CCMO) Register by as NL51648.091.14. Twenty patients with (recurrent) noninvasive vulvar Paget disease will be treated with topical 5% imiquimod cream three times a week for 16 weeks. The primary efficacy outcome is the reduction in lesion size at 12 weeks after end of treatment. Secondary outcomes are safety, immunological response, and quality of life. Safety will be assessed by evaluation of adverse events and tolerability of treatment. To evaluate the immunological response, various immunological markers will be tested on biopsy specimens taken before, during, and after treatment. Quality of life will be assessed with three questionnaires taken before, during, and after treatment.

**Results:**

First results are expected in the summer of 2018.

**Trial Registration:**

ClinicalTrials.gov NCT02385188; https://clinicaltrials.gov/ct2/show/NCT02385188 (Archived by WebCite at http://www.webcitation.org/6sXygHuhP).

## Introduction

Cutaneous Paget disease was first described in a series of patients with nipple ulceration and an underlying breast carcinoma. This became known as mammary Paget disease (MPD) [1]. When the same condition was reported on the scrotum and vulva, these were named extramammary Paget disease (EMPD) [2,3].

The presence of so-called Paget cells in the basal layers of the epithelium is pathognomonic for this rare disease. The origin of these large cells with abundant clear, pale cytoplasm, which often contain mucin, remains unclear. The most common hypothesis is that Paget cells originate from adnexal structures, such as apocrine glands or multipotent stem cells in the basal layer of the epidermis [4,5]. Other theories suggest the anogenital area contains mammary-like glands or that Toker cells, also seen in the nipple in mammary Paget disease, are precursor cells for EMPD [6-8].

The incidence rate of EMPD is 0.11 per 100,000 person-years, based on an epidemiological study with data of the Netherlands Cancer Registry [9]. Vulvar Paget disease (VPD) causes pain, itching, or a burning sensation, and a skin lesion, which can be described as a scaling, erythematous plaque that sometimes shows ulceration. VPD typically presents in postmenopausal Caucasian women [8].

Vulvar Paget disease can be divided into primary VPD, which is cutaneous, and secondary VPD, which is noncutaneous. Textbox 1 illustrates the different types of VPD [10].

Vulval Paget disease is associated with different malignancies, mainly an underlying vulvar, intestinal, or urological malignancy, and breast cancer. Approximately 20% of patients are reported to have an associated malignancy in their history. Therefore, screening for underlying carcinoma is advised, although there is no evidence for screening and no consensus on the extent of the additional diagnostic procedures [11,12].

Historically, the treatment of choice for VPD is wide local excision with clear margins, which is not always easy to realize on the vulva. Because Paget cells are found widely spread throughout the anogenital area, it is almost impossible to obtain clear surgical margins [13,14]. The recurrence rates of VPD are high: 15% to 70% independent of margin status. The risk of recurrence is highest in the first year after treatment [15]. To improve obtaining clear surgical margins, Mohs microsurgery has been evaluated for treatment of VPD. In Mohs microsurgery, the lesion is excised and the entire margin is examined immediately [16]. In case the margin is not clear, the excision is repeated, enlarging the circumference until the margins are clear. This technique may lead to lower recurrence rates [17]. However, large vulvar excisions may require plastic reconstruction.

Extensive vulvar surgery can cause permanent mutilation and functional impairment [18-22]. To address this problem, alternative treatment options such as photodynamic therapy, radiotherapy, chemotherapy, laser treatment, and recently topical 5% imiquimod cream have been used in patients with VPD with varying degrees of success [23-30].

Topical 5% imiquimod cream is an immune response modifier. It binds to toll-like receptor 7, inducing an innate and cell-mediated immune response [31]. It has antiviral and antitumor properties and is registered for the treatment of condylomata acuminata, actinic keratosis, and superficial basal cell carcinomas. Imiquimod also has shown to be effective for human papilloma virus-induced usual vulvar intraepithelial neoplasia [32,33]. The mechanism of action of imiquimod and local immunity in VPD are not known.

**Different types of vulvar Paget disease.**

**Primary EMPD (cutaneous)**

Type 1a: associated with noninvasive, intraepithelial disease

Type 1b: associated with invasive disease

Type 1c: associated with an underlying adenocarcinoma

**Secondary EMPD (noncutaneous)**

Type 2: EMPD originates from intestinal adenocarcinoma

Type 3: EMPD originates from urothelial carcinoma

More recently, a number of case reports, case series, and one observational trial that reported on the use of topical 5% imiquimod cream for VPD showed that imiquimod may be an effective treatment option [34,35]. A systematic review also concluded it is an effective alternative for VPD [36]. However, most studies described limited numbers of patients, various treatment schedules, and short follow-up periods. Therefore, it is impossible to pool data from previous studies to make final conclusions about the efficacy. The authors of the systematic review also mentioned the risk of publication bias: only positive results may be published retrospectively [36].

### Objective

The objective of this study is to assess the clinical efficacy, safety, and local immunity of topical 5% imiquimod cream in patients with noninvasive VPD.

## Methods

### Study Design

This study is a multicenter, prospective, open-label observational cohort study in patients with histologically proven, noninvasive VPD. Patients will be treated with topical 5% imiquimod cream three times a week for 16 weeks, with follow-up of one year after the end of treatment.

### Study Setting

Because VPD is rare, with an estimated incidence of four to seven cases per year in the Netherlands, the trial will be carried out in seven tertiary referral hospitals with a vulvar clinic in the Netherlands. Vulvar clinics are outpatient multidisciplinary clinics with participation of both gynecologists and dermatologists who are specialized in disorders of the vulva.

Participating centers are Antoni van Leeuwenhoek, Netherlands Cancer Institute, Amsterdam; Catharina Ziekenhuis, Eindhoven; Erasmus Medical Center, Rotterdam; Leiden University Medical Center; Radboudumc, Nijmegen; University Medical Center Groningen; and University Medical Center Utrecht.

### Participants

All patients with histologically proven noninvasive cutaneous VPD visiting or referred to a participating clinic will be asked to participate in this study. We estimate to include one patient per center per year because of the rarity of the disease.

Inclusion criteria are noninvasive VPD (primary or recurrence after earlier surgery or imiquimod treatment more than 6 months previously), age 18 years and older, and willing and able to comply with the protocol and provide informed consent in accordance with institutional and regulatory guidelines. Most patients are expected to be elderly, postmenopausal women, who may suffer from comorbidities. All patients will be instructed on how to apply the imiquimod cream by their clinician, according to the leaflet provided by the manufacturer, and using a mirror. If the patient is physically unable to apply the cream, a health care provider (eg, a nurse at the nursing home or via domiciliary care) will receive written instructions. If the patient consents, a printed photograph in which the affected skin is marked will be provided. Main exclusion criteria are current invasive VPD, underlying adenocarcinoma, and treatment of the vulva with topical 5% imiquimod cream during the last 6 months.

### Sample Size

Based on the estimated incidence of VPD in the Netherlands, viability is set at 20 inclusions. Our sample size considerations are based on the response rate. The primary outcome variable is the response at 12 weeks after end of treatment with topical 5% imiquimod cream. The only observational study on this topic, at time of conception of this trial, reported a response in 9 of 10 women [35]. Assuming a complete response rate of 80%, a cohort size of 20 patients is sufficient to estimate the complete response rate with a standard error of 9%, using the normal approximation for the binomial distribution. Because we presume that the dropout rate will not exceed 20%, a maximum of 25 patients will be included. When 20 patients have been treated with topical 5% imiquimod cream for at least 8 weeks, we will stop recruitment.

### Study Intervention

All patients will be treated with topical 5% imiquimod cream three times a week for 16 weeks. This treatment schedule is based on the treatment schedule for condylomata acuminata and on a previous randomized controlled trial of imiquimod 5% for usual vulvar intraepithelial neoplasia [31,33]. The healthy skin around the visible lesion can be protected with an indifferent basic ointment. Patients are allowed to use topical 3% lidocaine in Vaseline ointment if they experience pain at the application site. There must be a 1-hour interval between the application of different topical agents. Patients are also allowed to use paracetamol. In case of severe pain, when paracetamol and 3% lidocaine ointment are insufficient, it is permitted after consultation with the clinician to stop the treatment with topical 5% imiquimod cream for one week at a time. Patients are allowed to stop/delay treatment for a total of 3 weeks within the assigned treatment period.

**Table 1:** Study schedule presenting an overview of all study activities.

Study activities		0 w	Baseline	4 w	10 w	16 w	28 w	40 w	52 w	68 w
**Before treatment**										
	Mapping and mammography	X								
	Written informed consent	X								
	Require histological samples (incl. mapping) and mammography results from referring hospital	X								
**Imiquimod 5% cream**			Start			Stop				
	Consultation by phone				X					
	Consultation at outpatient clinic of involved research clinic, containing:		X	X		X	X	X	X	X
	Vulvar examination, measurement and photo documentation		X	X			X			X
	VAS score		X	X	X	X	X	X	X	X
	Tolerability questionnaire			X	X	X				
	Biopsy	X		X			X			
	EQ5D		X	X			X			
	DLQI		X	X			X			
	FSDS		X	X			X			
	Review patient diary			X	X	X				

In case of a suspected secondary bacterial infection, fucidin cream or ointment 20 mg/g will be prescribed. The patient will apply the fucidin cream or ointment three times a day, according to the prescription. No other local products than imiquimod cream, lidocaine, indifferent moisturizers, or fucidin are allowed to be applied at the lesion site. On an individual basis, other topical products will be considered as a protocol violation.

### Study Schedule

Patients will visit the clinic seven times during the study; the final visit will be 1 year after the end of treatment (Table 1). One consultation will take place by telephone. During these consultations, pain will be measured by means of the visual analog scale (VAS) score for pain. Pain, burning, and itching will be asked on a four-point Likert scale. During the visits, the clinical response will be evaluated by vulvar examination and bidimensional measurement of the visible lesions. The histological effect will be assessed by pathological assessment of the presence of Paget cells in the biopsy sample(s) taken 12 weeks after the end of treatment. All biopsy samples taken before, during, and after treatment will be taken around the same location. The site of the first biopsy is most likely the most evident lesion, causing a clinically visible lesion. The site of this biopsy will be recorded in the case report file to ensure other biopsies will be taken at the same area. Quality of life will be assessed before, during, and after treatment using three questionnaires on general health (EQ-5D), dermatological quality of life (Dermatology Quality of Life Index [DLQI]), and (if applicable) sexual functioning (Female Sexual Distress Scale [FSDS]).

Safety will be evaluated by documentation of all adverse effects, recorded by the clinician and by the patient in the patient diary.

The immunological effect will be assessed by comparing the results of additional immunohistochemistry stains performed on all three samples taken around the same location at baseline, 4 weeks after start of treatment, and 12 weeks after end of treatment. All biopsies will be taken at approximately the same location to ensure the local microenvironment is as similar as possible in all samples. There are limited data on the tumor microenvironment in VPD; we are currently performing a separate pilot study to investigate the parameters in the immune infiltrate in VPD. We are investigating which immune cells are present in VPD, and will use this knowledge to further explore which immune cells respond to the topical imiquimod cream and the role they play in the origin and treatment of VPD. The results of this separate pilot study will be used to decide which markers will be investigated in the samples collected in the Paget Trial.

### Study Endpoints

The main study outcome is clinical response. This will be assessed by determination of the reduction in lesion size 12 weeks after the end of treatment. This will ensure any local skin effects caused by treatment will be healed at time of examination. All measurements during the study will be conducted by the same trained and experienced local clinician. Photographs for documentation will be taken with a ruler alongside the lesions. The comparison between the lesion size at the start of treatment and 12 weeks after the end of treatment can lead to the following outcomes:

1. Complete response: defined as disappearance of the lesion and histological confirmation of disappearance;
2. Partial response: defined as decrease by ≥50% of total lesion size;
3. No response: defined as <50% decrease of total lesion size; or
4. Progressive disease: defined as ≥25% increase of total lesion size or progression into invasive disease and/or adenocarcinoma.

Secondary outcomes are the safety, quality of life, and the assessment of local immunological response. These outcomes will be assessed according to the following criteria:

1. Safety: all adverse events that occur during the study will be collected by the clinician at every consultation (at the clinic or via telephone) and by the patient using a standardized patient diary.
2. Quality of life: results of the three questionnaires (EQ-5D, DLQI, and, if applicable, FSDS) taken before, during, and after treatment will be compared.

Local immunological response will be assessed by a set of markers, to be determined, in tissue samples obtained by vulvar biopsy before, during, and after treatment.

### Statistical Analysis:

An intention-to-treat (ITT) and per protocol (PP) analysis will be performed. The population included in the ITT analysis is defined as all patients that have started treatment with topical 5% imiquimod cream. The PP analysis will include patients that have completed treatment with topical 5% imiquimod cream according to protocol. Two-tailed *P* values <.05 will be considered statistically significant. Our primary study parameter is the clinical response to topical 5% imiquimod cream. Twelve weeks after the end of treatment, the clinician will examine the vulva of the patient and assign the patient in one of the response categories as defined previously. Estimates of the percentage responders per response category will be presented with corresponding 95% confidence intervals. The relation between treatment duration and dose versus response will be explored. Safety will be analyzed in a descriptive manner, presenting all adverse events (local and systemic) in all participants treated with topical 5% imiquimod cream. Also, the use of painkillers, lidocaine ointment, and discontinuation of treatment will be reported.

Quality of life will be assessed by three questionnaires. The EQ-5D results will be converted to the crosswalk index values, using the Crosswalk Index Value Calculator [37]. The DLQI results will be categorized according to the instruction manual, ranging from “no effect at all on patient’s life” to “extremely large effect on patient’s life” [38]. The result of the FSDS is the sum of the answers. Descriptive statistics will be used to present the change outcomes during treatment versus before treatment, and after treatment versus before treatment. A subanalysis of responders and nonresponders will be conducted.

The immunological results will be counted and compared between the different biopsy samples. These data will be reported in a descriptive manner.

### Ethics

This study will be conducted according to the principles of the Declaration of Helsinki (2008) and the Medical Research Involving Human Subjects Act (Dutch: WMO). The protocol has been medical-ethically approved by the Medical-Ethical Committee of Arnhem-Nijmegen to be conducted in all seven centers (NL51648.091.14). Before enrollment to the study, written informed consent will be obtained from all patients.

## Results

The study opened for enrollment in January 2015. Currently, 17 patients are participating in this trial. The first results are expected in the summer of 2018.

## Discussion

Currently, this study is the first prospective study examining the clinical efficacy of topical 5% imiquimod cream in patients with noninvasive VPD using a standardized treatment schedule over 16 weeks. In addition, this study will also be the first to investigate the safety, quality of life, and immunological response of 5% imiquimod cream therapy in patients with VPD.

Until now, about 25 retrospective case series have been published on this topic. These studies show high success rates. The effectiveness of topical 5% imiquimod cream for VPD in these cases might be overrated due to publication bias in these retrospective cases. Most of the retrospective series have used different treatment schedules. The prospective trial of Marchitelli et al [35] used a different treatment schedule per patient. In most case studies, treatment was continued until the patient obtained a complete response. The pilot study by Cowan et al [34] investigated the clinical response after 12 weeks of treatment in eight patients with noninvasive VPD. Patients applied the cream three times a week. Six patients had a clinical and histological complete response; the other two had a partial response with histological persistence. In our study, all 20 consecutive patients will be treated according to the same treatment schedule: three times a week for 16 weeks. Currently, there are no guidelines for topical 5% imiquimod treatment for VPD. We based the treatment schedule on the treatment schedule for condylomata acuminata because this is a registered indication and therefore we consider this treatment schedule to be safe for genital skin [31]. Furthermore, VPD may be considered a vulvar premalignancy, and the same treatment schedule is used in a previous randomized controlled trial of imiquimod 5% for usual vulvar intraepithelial neoplasia [33].

There are very limited data concerning the influence of VPD on everyday life of the patient. It is reported that vulvar surgery may contribute to decreased quality of life and sexual functioning compared to healthy patients. As VPD has high recurrence rates, we assume (repetitive) surgical treatment may have significant psychosexual effects on patients. Topical treatment with 5% imiquimod cream will not induce scarring nor will it alter the anatomy of the vulva. Because there is a lack of data on this specific topic, we will investigate quality of life with three different questionnaires before, during, and after treatment.

The mechanism of action and immunological effects of 5% imiquimod cream in VPD are uncertain. It is likely that imiquimods’ immune modulating effect induces a local immune response resulting in clearance of the Paget cells. Investigating the immunological response in biopsy specimens taken before, during, and after treatment will provide insight in the local effects of imiquimod in the skin and also in the underlying mechanisms of action. Unfortunately, there is no current literature on this topic. Therefore, we are conducting a pilot study, investigating the microenvironment of VPD, to assess which markers may be valuable in understanding the immunological response in VPD.

In conclusion, VPD remains an elusive disease. Surgery has been the treatment of choice for over a century. Due to high recurrence rates and the vulnerable patient population affected by the disease, there is a need for other less-invasive treatment options. Topical 5% imiquimod cream may be an attractive alternative. Our trial will investigate the clinical efficacy of topical 5% imiquimod cream in 20 patients with a standardized treatment schedule. This study will also evaluate the safety, quality of life, and immunological response while using 5% imiquimod cream.

## Abbreviations

DLQI: Dermatology Quality of Life Index
EMPD: extramammary Paget disease
FSDS: Female Sexual Distress Scale
ITT: intention to treat
MPD: mammary Paget disease
PP: per protocol
VAS: visual analog scale
VPD: vulvar Paget disease

## References

[1] Paget J (1874). On disease of the mammary areola preceding cancer of the mammary gland. St Bartholemew Hosp Res Lond.

[2] Dubreuilh W (1901). Paget's disease of the vulva. Br J Dermatol.

[3] Crocker H (1889). Paget's disease affecting the scrotum and penis. T Pathological Soc.

[4] Finan MA, Barre G (2003). Bartholin's gland carcinoma, malignant melanoma and other rare tumours of the vulva. Best Pract Res Clin Obstet Gynaecol.

[5] Kneale BL, Fortune DW (1991). Pathology of the vulva. Curr Opin Obstet Gynecol.

[6] Belousova IE, Kazakov DV, Michal M, Suster S (2006). Vulvar toker cells: the long-awaited missing link: a proposal for an origin-based histogenetic classification of extramammary paget disease. Am J Dermatopathol.

[7] Willman JH, Golitz LE, Fitzpatrick JE (2005). Vulvar clear cells of Toker: precursors of extramammary Paget's disease. Am J Dermatopathol.

[8] Kazakov DV, Spagnolo DV, Kacerovska D, Michal M (2011). Lesions of anogenital mammary-like glands: an update. Adv Anat Pathol.

[9] Siesling S, Elferink MA, van Dijck JA, Pierie JP, Blokx WA (2007). Epidemiology and treatment of extramammary Paget disease in the Netherlands. Eur J Surg Oncol.

[10] Wilkinson E, Brown H (2002). Vulvar Paget disease of urothelial origin: a report of three cases and a proposed classification of vulvar Paget disease. Hum Pathol.

[11] Mendivil AA, Abaid L, Epstein HD, Rettenmaier MA, Brown JV, Micha JP, Wabe MA, Goldstein BH (2012). Paget's disease of the vulva: a clinicopathologic institutional review. Int J Clin Oncol.

[12] Delport ES (2013). Extramammary Paget's disease of the vulva: an annotated review of the current literature. Australas J Dermatol.

[13] Baehrendtz H, Einhorn N, Pettersson F, Silfverswärd C (1994). Paget's disease of the vulva: the Radiumhemmet series 1975-1990. Int J Gynecol Cancer.

[14] Gunn RA, Gallager HS (1980). Vulvar Paget's disease: a topographic study. Cancer.

[15] van der Zwan JM, Siesling S, Blokx WA, Pierie JPE, Capocaccia R (2012). Invasive extramammary Paget's disease and the risk for secondary tumours in Europe. Eur J Surg Oncol.

[16] Mohs FE, Blanchard L (1979). Microscopically controlled surgery for extramammary Paget's disease. Arch Dermatol.

[17] Bae JM, Choi YY, Kim H, Oh BH, Ro MR, Nam K, Chung KY (2013). Mohs micrographic surgery for extramammary Paget disease: a pooled analysis of individual patient data. J Am Acad Dermatol.

[18] Fanning J, Lambert L, Hale TM, Morris PC, Schuerch C (1999). Paget's disease of the vulva: prevalence of associated vulvar adenocarcinoma, invasive Paget?s disease, and recurrence after surgical excision. Am J Obstet Gynecol.

[19] Funaro D, Krasny M, Lam C, Desy D, Sauthier P, Bouffard D (2013). Extramammary Paget disease: epidemiology and association to cancer in a Quebec-based population. J Low Genit Tract Dis.

[20] Chan J, Li GK, Chung JH, Chow VL (2012). Extramammary Paget's disease: 20 years of experience in Chinese population. Int J Surg Oncol.

[21] Black D, Tornos C, Soslow RA, Awtrey CS, Barakat RR, Chi DS (2007). The outcomes of patients with positive margins after excision for intraepithelial Paget's disease of the vulva. Gynecol Oncol.

[22] Shaco-Levy R, Bean SM, Vollmer RT, Papalas JA, Bentley RC, Selim MA, Robboy SJ (2010). Paget disease of the vulva: a histologic study of 56 cases correlating pathologic features and disease course. Int J Gynecol Pathol.

[23] Al Yousef A, Boccara O, Moyal-Barracco M, Zimmermann U, Saiag P (2012). Incomplete efficacy of 5-aminolevulinic acid (5 ALA) photodynamic therapy in the treatment of widespread extramammary Paget's disease. Photodermatol Photoimmunol Photomed.

[24] Raspagliesi F, Fontanelli R, Rossi G, Ditto A, Solima E, Hanozet F, Kusamura S (2006). Photodynamic therapy using a methyl ester of 5-aminolevulinic acid in recurrent Paget's disease of the vulva: a pilot study. Gynecol Oncol.

[25] Karam A, Dorigo O (2012). Treatment outcomes in a large cohort of patients with invasive Extramammary Paget's disease. Gynecol Oncol.

[26] Son SH, Lee JS, Kim YS, Ryu MR, Chung SM, Namkoong SE, Han GT, Lee HJ, Yoon SC (2005). The role of radiation therapy for the extramammary paget's disease of the vulva; experience of 3 cases. Cancer Res Treat.

[27] Hanawa F, Inozume T, Harada K, Kawamura T, Shibagaki N, Shimada S (2011). A case of metastatic extramammary Paget's disease responding to trastuzumab plus paclitaxel combination therapy. Case Rep Dermatol.

[28] Tauveron V, Body G, Machet L, Lenain H, Ouldamer L, Lorette G (2014). Prolonged remission of Paget disease of the vulva after chemotherapy for breast carcinoma. Br J Dermatol.

[29] Ewing TL (1991). Paget's disease of the vulva treated by combined surgery and laser. Gynecol Oncol.

[30] Valentine BH, Arena B, Green E (1992). Laser ablation of recurrent Paget's disease of vulva and perineum. J Gynecol Surg.

[31] European Medicines Agency (2008). European Public Assessment Report (EPAR): Aldara.

[32] Terlou A, van Seters M, Kleinjan A, Heijmans-Antonissen C, Santegoets LA, Beckmann I, van Beurden M, Helmerhorst TJ, Blok LJ (2010). Imiquimod-induced clearance of HPV is associated with normalization of immune cell counts in usual type vulvar intraepithelial neoplasia. Int J Cancer.

[33] van Seters M, van Beurden M, ten Kate FJ, Beckmann I, Ewing PC, Eijkemans MJ, Kagie MJ, Meijer CJ, Aaronson NK, Kleinjan A, Heijmans-Antonissen C, Zijlstra FJ, Burger MP, Helmerhorst TJ (2008). Treatment of vulvar intraepithelial neoplasia with topical imiquimod. N Engl J Med.

[34] Cowan RA, Black DR, Hoang LN, Park KJ, Soslow RA, Backes FJ, Gardner GJ, Abu-Rustum NR, Leitao MM, Eisenhauer EL, Chi DS (2016). A pilot study of topical imiquimod therapy for the treatment of recurrent extramammary Paget's disease. Gynecol Oncol.

[35] Marchitelli C, Peremateu MS, Sluga MC, Berasategui MT, Lopez DG, Wernicke A, Velazco A, Gogorza S (2014). Treatment of primary vulvar paget disease with 5% imiquimod cream. J Low Genit Tract Dis.

[36] Machida H, Moeini A, Roman Ld, Matsuo K (2015). Effects of imiquimod on vulvar Paget's disease: a systematic review of literature. Gynecol Oncol.

[37] EQ-5D.

[38] Cardiff University Department of Dermatology.

